# Adiabatic Generation of N-quNit Singlet States with Cavity QED

**DOI:** 10.1038/srep45756

**Published:** 2017-04-03

**Authors:** Rong-Can Yang, Li-Xiang Ye, Xiu Lin, Hong-Yu Liu

**Affiliations:** 1 Fujian Provincial Key Laboratory of Quantum Manipulation and New Energy Materials, and College of Physics and Energy, Fujian Normal University, Fuzhou, 350007, China; 2 Fujian Provincial Collaborative Innovation Center for Optoelectronic Semiconductors and Efficient Devices, Xiamen 361005, China; 3 School of Electrical, Computer, and Energy Engineering, Arizona State University, Tempe, Arizona 85287, USA; 4 College of Science, Yanbian University, Yanji, 133002, China

## Abstract

We present a theoretical scheme to generate N-quNit singlet states with *N* 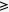 3 via adiabatic passage. In this protocol, the system may be robust against both experimental parameter fluctuations and dissipations along dark states. In addition, during the whole procedure, quantum information is almost fully transferred between atomic ground states. It reduces the influence of dissipations such as atomic spontaneous emissions and cavity decays. Thus, the presented proposal may be feasible based on current technologies.

Quantum entanglement, originally proposed by Einstein *et al*.^1^, plays a central role in the test of Bell’s theorem without inequalities^2^. It now has become an important resource for quantum information processing, quantum computation, quantum measurement, and so on^3^,^4^,^5^,^6^,^7^,^8^. Quantum entanglement can be divided into several categories mainly according to two parameters, e.g., subsystems’ numbers and dimensions. But even when the two parameters are same, quantum structure may be different, such as W and GHZ states for entanglement among three qubits^9^. It is now known that different types possess their own distinct properties in quantum society. Especially for multi-level (or called high-dimension) entangled states, they are more significant in testing quantum nonlocality and realizing quantum informaiton processing than entangled qubits due to their richer structures^2^,^10^,^11^. Thereby, there are lots of documents to discuss the preparation and applications of multi-level entanglement. In this paper, we focus on a special one, i.e. a singlet state. It was put forward by Cabello in 2002 and considered to be robust against collective decoherence due to their total spins being equal to zero^12^, which makes it attractive. In other words, any an entangled state with total spin zero is called singlet states. So, singlet states involve several types. Here, what we are interested in is the N-particle-and-N-dimension singlet state, (i.e. N-quNit singlet state, or NNSS for short) whose distinctive characteristic is to realize an unknown unitary transformation^13^ and to solve several practical problems without classical solutions, including “N -strangers”, “secret sharing” and “liar detection”^14^. An NNSS can be mathematically expressed as


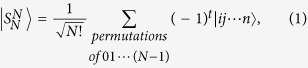


where *t* is the number of transpositions of pairs of elements that must be composed to place the elements in canonical order.

Although singlet states may be significant for quantum computation, it is a great challenge to prepare NNSS when 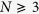 not only in theory but also in experiment. The reason is that it is hard to find a system including N particles with N dimensions and let them interact simultaneously. Until 2005, Jin *et al*. presented a scheme for the preparation of NNSS with *N* = 3 (33SS), attracting much attention^15^. After that, several scenarios were proposed for generating 33SS^16^,^17^,^18^,^19^,^20^,^21^,^22^,^23^, in which only two different methods were used. One method needs several steps to generate 33SS^15^, while the other one proposed by us needs only one step to generate 33SS from 22SS^18^ and it may be generalized for generating NNSS with *N* > 3 more easily than the first one. Based on the second method, Shao *et al*. suggested a model for a series of Λ-like multilevel atoms interacting with a multi-mode cavity in 2010^24^. However, in their work, high-dimension NNSS with *N* > 3 may be hardly realized because it need all atoms to interact with a (N − 1)-mode cavity. In 2016, the similar idea was discussed by Chen *et al*.^25^. Can we avoid the use of a multi-mode cavity for the generation of NNSS? Yes. An alternative scheme using Rydberg-blockade mechanism and adiabatic-passage technology was reported by us also in 2016^26^. But the scheme may be strongly influenced by spontaneous emission of ladder-type Rydberg atoms.

How can we realize storing an NNSS in ground states and avoid the use of a multi-mode cavity at the same time? In this paper, we suggest to generate NNSS for atoms with *N* − 1 excited states and *N* ground states in a single-mode cavity via adiabatic passage. The reason why we use adiabatic-passage technology is that it is insensitive to the variation of experimental parameters^27^,^28^,^29^. Based on this technology or its further versions (such as transitionless quantum driving^30^ and invariant-based shortcut^31^), many suggestions are shown to realize multi-level entanglement, e.g. shown in the refs 22, 23, 25, 26 and 32.

This paper is organized as follows. Section II describes how to realize a 33SS on the basis of 22SS. Section III introduces how to generate NNSS. In Section IV, some numerical simulations and analysis are made to show the feasibility of the preparation of NNSS. Finally, we draw a conclusion is made in Section V.

## Generation of atomic 33SS

**Figure 1:**
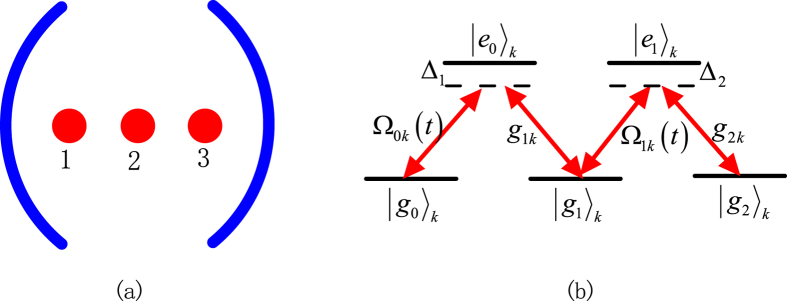
Schematic diagram for the generation of 33SS. (**a**) Atoms in a single-mode cavity; (**b**) Atomic level configurations.

Figure 1 illustrates three atoms are trapped in a single-mode cavity. Each atom has three ground states |*g*_0_〉_*k*_, |*g*_1_〉_*k*_, |*g*_2_〉_*k*_ and two excited states |*e*_0_〉_*k*_, |*e*_1_〉_*k*_ with the subscript *k* representing the *k*th atom. The *j*th (*j* = 0, 1) laser pulse focuses on the transition 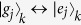 with time-dependent Rabi frequency Ω_*jk*_(*t*) and frequency detuning Δ_*j*+1_, while the transition 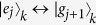 is coupled to the cavity with coupling constant *g*_(*j*+1)*k*_ and the frequency detuning Δ_*j*+1_, meaning that each two adjacent ground states for each atom interact with laser and the cavity mode by the two-photon resonance. With the consideration of resolved sideband and rotating-wave approximation, the total Hamiltonian for this system, in the interaction picture, reads (*ħ* = 1)


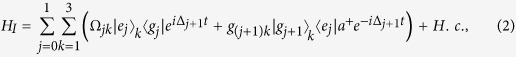


where *a*^+^ is a creation operator of the cavity mode and *H.c*. denotes Hermitian conjugate terms.

Taking into account of large detuning conditions, i.e. 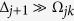, 
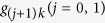
, we can adiabatically eliminate the excited states |*e*_*j*_〉_*k*_, leading the interaction Hamiltonian to be


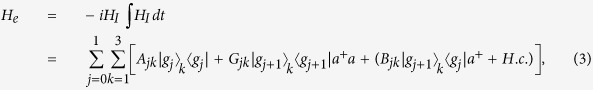


with


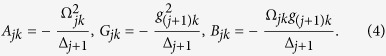


The first term describes Stark shifts induced by classical lasers, the second one shows photon-number-dependent Stark shifts induced by the cavity mode, and the third denotes the interaction between the cavity mode and the transition of the two ground states 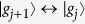.

For the sake of simplicity, we add another laser pulses for nonresonantly coupling to the atomic ground states so as to compensate atomic Stark shifts by laser pulses (the first term described in Eq. (3))^33^, then we have


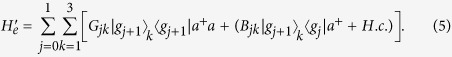


In addition, we suppose the second and the third atoms are identical, resulting in *X*_*j*3_ = *X*_*j*2_ = *X*_*j*_, where *X* = Ω, *g*, Δ, *A, B, G* and the Stark shifts for these three atoms induced by the cavity mode are equal, i.e. *G*_0*k*_ = *G*_1*k*_ = *G*. For the motivation to prepare 33SS, the system is initially in the state 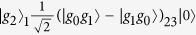, i.e. the first atom is initially in |*g*_0_〉, the second and the third ones are prepared in 22SS, and the cavity is in the vacuum state |0〉. To calculate the evolution of the system state and make it compact, we first define some states and operators:


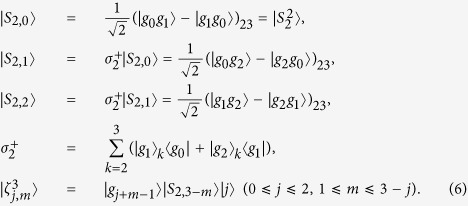


Then the whole system without consideration of any dissipations will be confined in the closed subspace 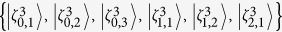, reducing the effective Hamiltonian (5) to be


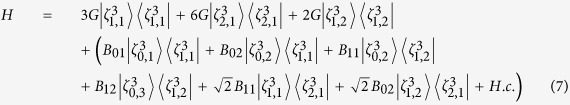


Reshaping the Eq. (7) in the matrix form and diagonalizing it, we can obtain a time-dependent dark state with a null eigenvalue, i.e.


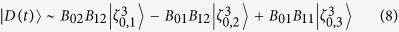


For simplicity, we set *B*_1*k*_ = *χ*_1_*B*_0*k*_(*t*) (*k* = 1, 2) with *χ*_1_ being time-independent. Due to 
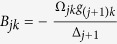
, if we design laser pulses satisfying^28^


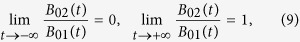


we can adiabatically transfer the initial system state 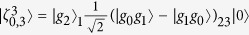 to a 33SS


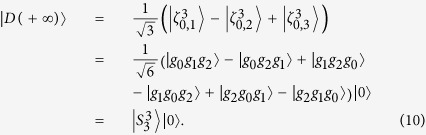


Now, we have prepared a 33SS on the basis of 22SS with the cavity mode always in the vacuum state.

## Generalization for the preparation of NNSS

**Figure 2:**
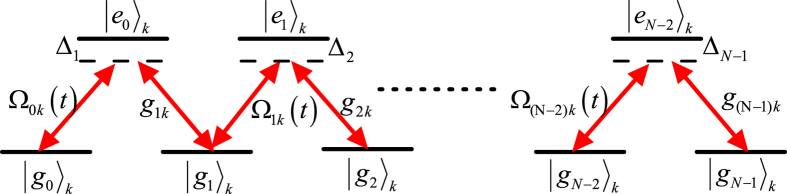
Atomic level structure for NNSS.

In this section, we generalize the above suggestion to prepare NNSS. Similarly, we need to trapped *N* atoms with *N* ground states and *N* − 1 excited states in a single-mode cavity. The atomic structure and transitions are shown in Fig. 2. We also consider the large-detuning condition to eliminate excited states and add another laser pulses to get rid of Stark shifts induced by laser pulses. For simplicity, we assume all atoms except the first one are identical and all Stark shifts induced by the cavity mode are same. In this situation, the effective Hamiltonian for these *N* atoms and the cavity mode is


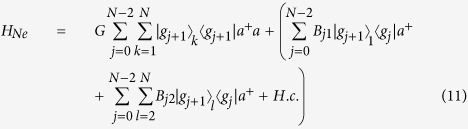


where *G* = *G*_*jk*_, 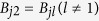 with *G*_*jk*_, *B*_*j*1_, *B*_*jl*_ being defined in Eq. (4). It should be pointed out although Stark shifts *G*_*jk*_ for all levels can not directly be equal, but we can use compensating technologies to let them be approximately equal, which is sufficient for our proposal. We assume that the system is initially prepared in the state 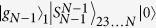, i.e. the initial state of the first atom (the other N − 1 atoms) is |*g*_*N*−1_〉 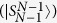, and the cavity mode is in the vacuum state |0〉. Similar to Eq. (6), let us define some states and operators for all atoms except the first one, i.e.


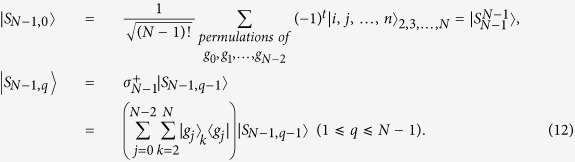


In the subspace


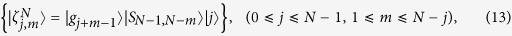


the Hamiltonian reduces to


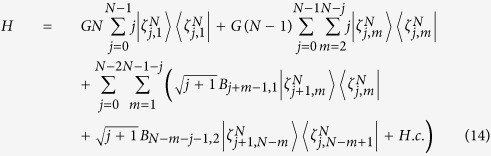


**Figure 3:**
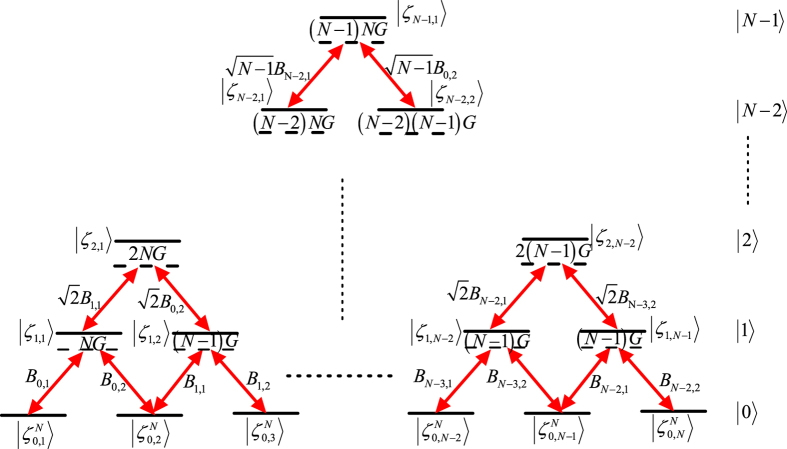
Logic states and transitions for generating NNSS. A black solid line represents a system logic state 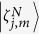 defined in Eq. (13), a black dashed line gives an illustration for the detuning, and 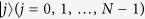 on the right side is to show all the left logic states share the same cavity field |*j*〉.

In order to demonstrate the relationship among logic states 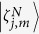 in the subspace and how to generalize the scheme to prepare NNSS with *N* > 3, we illustrate them in Fig. 3, where, for example, the lowest levels correspond to the system state with the cavity mode in the vacuum state. After a calculation to diagonalize the matrix described in Eq. (14), we found the system has a time-dependent dark state, i.e.


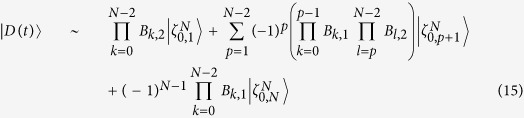


For simplicity, we set *B*_*l,s*_ = *χ*_*l*_*B*_0,*s*_(*t*) (*l* = 1, 2, …, *N* − 2; *s* = 1, 2) with *χ*_*l*_ being time-independent. Then we design laser pulses satisfying


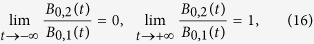


for adiabatic transfer from the initial system state 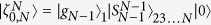 to an NNSS


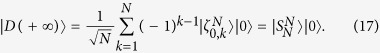


In this situation, we have prepared an NNSS with the cavity mode always in the vacuum state.

## Numerical analysis

**Figure 4:**
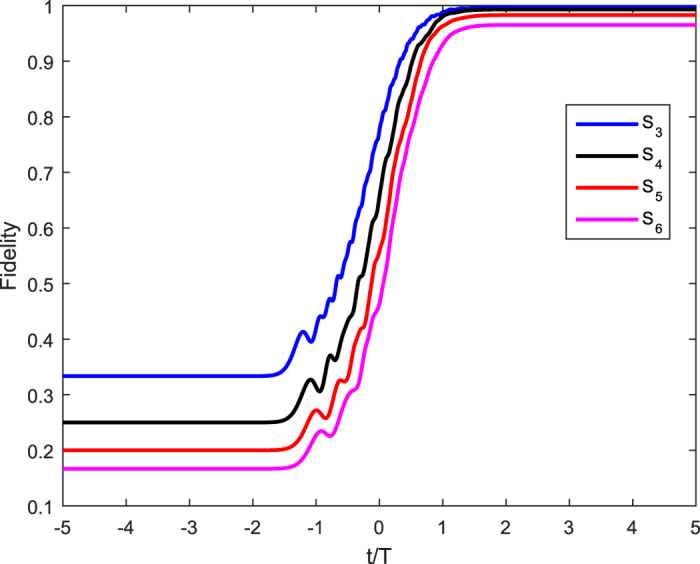
The function of the fidelity of the generated singlet states for *N* = 3, 4, 5, 6 versus the dimensionless interaction time *t/T* with *T* = 800/Ω_0_, *τ* = *T*/2, *g* = Ω_0_, Δ = 10 Ω_0_.

According to Eq. (16), we design two time-dependent pulses to be ref. 28


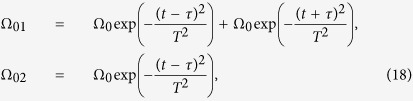


where Ω_0_ represents the time-independent amplitude and *T (τ*) the pulse width (delay) and then plot the fidelity 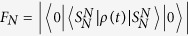 versus the variations of the interaction time t in Fig. 4, where the density matrix for the whole system *ρ(t*) satisfies the Schr*ö*dinger equation 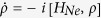 and *H*_*Ne*_ is shown in Eq. (11). In Fig. 4, we have only given the evolution for *N* = 3, 4, 5, 6 with the choice of 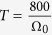, 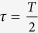, *g* = Ω_0_, Δ = 10 Ω_0_. It should be noted that there are similar results for larger *N*.

Results show that the fidelity for *N* = 3 to 5 can rise to the value close to 0.99, and the maximum fidelity for *N* = 6 can also reach about 0.97, meaning that a singlet state for three, four, five, or six atoms can be generated. And we can predict an atomic NNSS with 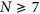 can be realized if we have prepared (N − 1) (N − 1)SS.

**Figure 5:**
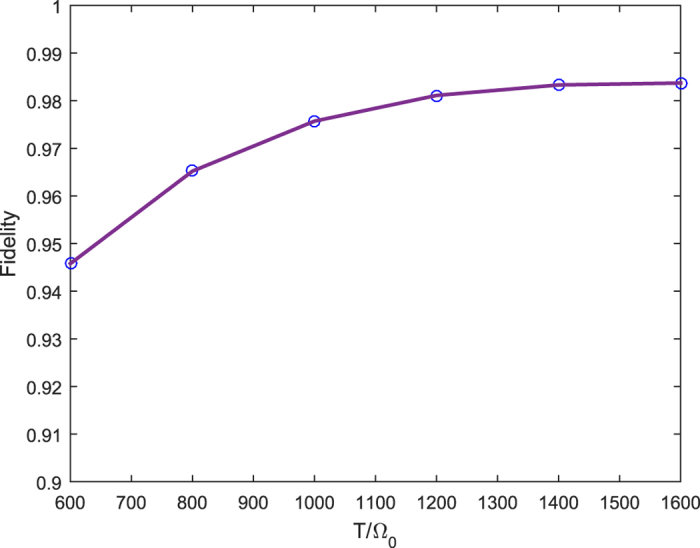
Fidelity of the 66SS versus pulse width, where *g* = Ω_0_, Δ = 10 Ω_0_, *τ* = *T*/2, *t* = 5*T*.

However, we should admit that the fidelity will decrease with the increase of *N* when all parameters have been set. For example, the fidelity for 55SS is about 0.99, whereas the fidelity of 66SS is only 0.97. Why does it happen? Here, we would show that the increase of pulse width, pulse delay and required time may make it happen. Figure 5 illustrates the fidelity of 66SS as a function of pulse width, where we set *g* = Ω_0_, Δ = 10 Ω_0_, 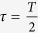, *t* = 5*T*. It was clearly demonstrated that without consideration of the dissipation, the larger *T* is, the greater the fidelity *F* is, and the fidelity will finally reach a stable value which is close to 1. For instance, the fidelity raises from *F* = 0.946 to 0.981 with the increase of *T* from 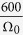 to 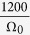. Although we may improve the fidelity in this way, we should also accept the fact that it may result in some other problems, such as the effect of photon decay and spontaneous emission. Therefore, our protocol may only be feasible for limited number of atoms.

**Figure 6:**
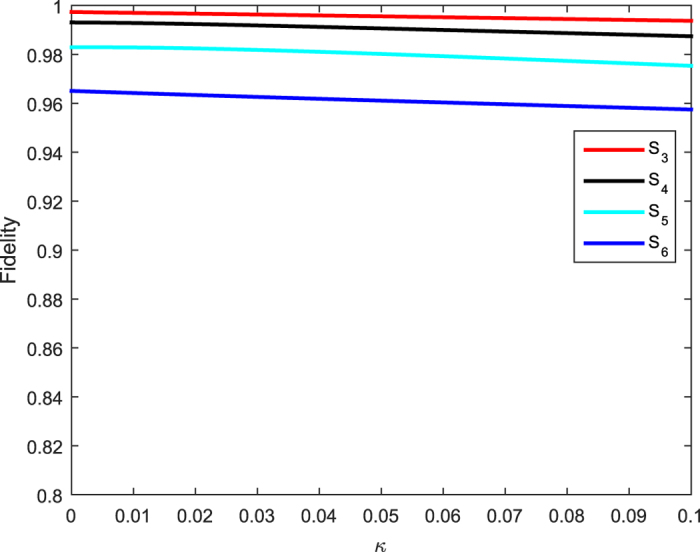
The fidelity of NNSS with *N* = 3, 4, 5, 6 versus the cavity decay reate *κ* with *g* = Ω_0_, Δ = 10 Ω_0_, *T* = 800/Ω_0_, *τ* = *T*/2, *t* = 5*T*.

Last but not the least, we take the effect of photon decay into account. We resort to the master equation expressed as


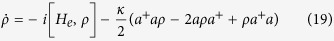


where *κ* denotes the decay rate of the cavity mode. In Fig. 6, we only depict the curve for the relationship of the fidelity versus the decay for 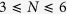, where we have set *g* = Ω_0_, Δ = Ω_0_, 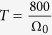, 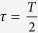, *t* = 5*T*. Figure 5 clearly shows that the fidelity of the generated singlet states is scarcely insensitive to cavity decay. For example, the fidelity for *N* = 3 only drops from *F* = 0.997 to 0.994 when the cavity decay rate increases from *κ* = 0 to 0.1, while the fidelity for *N* = 6 only drops from *F* = 0.965 to 0.957. It should be noticed that the system would be influenced by spontaneous emission of atoms, but this effect may be suppressed with all atoms only virtually excited.

## Feasibility and conclusions

In this section, we first make a brief discussion on the feasibility. The atomic levels used in our proposal can easily be found in hyperfine states of natural or artificial atoms, such as Cs atoms. Of course, the scale for the generated singlet states may be limited by atomic structures. For example, if we choose hyperfine states 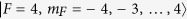 of 6^2^*S*_1/2_ of ^133^*Cs* atoms to act as ground states 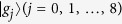 and 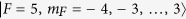 of 6^2^*P*_3/2_ to act as excited states 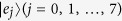, then we can only realize the generation of an N-quNit singlet state with *N* = 9 in an ideal condition. In addition, if we choose the parameters 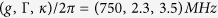 which is predicted by Spillane *et al*.^34^, then the fidelities for the generated singlet states for *N* = 3, 4, 5, 6 are about 0.997, 0.993, 0.983, 0.965, respectively. Hence, our proposal may be feasible for generating singlet states of the limited number N of atoms.

In summary, a novel scheme has been proposed to generate NNSS with adiabatic passage, which may be suitable for arbitrary *N* in theory. Although the scale of a singlet state in the paper is restricted by atomic structures, it may open up a new idea for the generation of NNSS. In our scheme, quantum information is only transferred among atomic ground states, leading the system to be insensitive to atomic spontaneous emission and photon decays. In conclusion, the proposed scheme may be implemented with current techniques.

## Additional Information

**How to cite this article:** Yang, R.-C. *et al*. Adiabatic Generation of N-quNit Singlet States with Cavity QED. *Sci. Rep.* **7**, 45756; doi: 10.1038/srep45756 (2017).

**Publisher's note:** Springer Nature remains neutral with regard to jurisdictional claims in published maps and institutional affiliations.
